# Quantitative *in vivo* mapping of myocardial mitochondrial membrane potential

**DOI:** 10.1371/journal.pone.0190968

**Published:** 2018-01-16

**Authors:** Nathaniel M. Alpert, Nicolas Guehl, Leon Ptaszek, Matthieu Pelletier-Galarneau, Jeremy Ruskin, Moussa C. Mansour, Dustin Wooten, Chao Ma, Kazue Takahashi, Yun Zhou, Timothy M. Shoup, Marc D. Normandin, Georges El Fakhri

**Affiliations:** 1 Gordon Center for Medical Imaging, Massachusetts General Hospital, Harvard Medical School, Boston, Massachusetts, United States of America; 2 Cardiac Arrhythmia Service, Massachusetts General Hospital, Harvard Medical School, Boston, Massachusetts, United States of America; 3 The Russell H. Morgan Department of Radiology and Radiological Science, School of Medicine Johns Hopkins University, Baltimore, Maryland, United States of America; Instituto Nacional de Cardiologia Ignacio Chavez, MEXICO

## Abstract

**Background:**

Mitochondrial membrane potential (*ΔΨ*_*m*_) arises from normal function of the electron transport chain. Maintenance of *ΔΨ*_*m*_ within a narrow range is essential for mitochondrial function. Methods for in vivo measurement of *ΔΨ*_*m*_ do not exist. We use ^18^F-labeled tetraphenylphosphonium (^18^F-TPP^+^) to measure and map the total membrane potential, ΔΨ_T_, as the sum of *ΔΨ*_*m*_ and cellular (*ΔΨ*_*c*_) electrical potentials.

**Methods:**

Eight pigs, five controls and three with a scar-like injury, were studied. Pigs were studied with a dynamic PET scanning protocol to measure ^18^F-TPP^+^ volume of distribution, V_T_. Fractional extracellular space (*f*_*ECS*_) was measured in 3 pigs. We derived equations expressing ΔΨ_T_ as a function of V_T_ and the volume-fractions of mitochondria and *f*_*ECS*_. Seventeen segment polar maps and parametric images of ΔΨ_T_ were calculated in millivolts (mV).

**Results:**

In controls, mean segmental ΔΨ_T_ = -129.4±1.4 mV (SEM). In pigs with segmental tissue injury, ΔΨ_T_ was clearly separated from control segments but variable, in the range -100 to 0 mV. The quality of ΔΨ_T_ maps was excellent, with low noise and good resolution. Measurements of ΔΨ_T_ in the left ventricle of pigs agree with previous in in-vitro measurements.

**Conclusions:**

We have analyzed the factors affecting the uptake of voltage sensing tracers and developed a minimally invasive method for mapping ΔΨ_T_ in left ventricular myocardium of pigs. *ΔΨ*_*T*_ is computed in absolute units, allowing for visual and statistical comparison of individual values with normative data. These studies demonstrate the first in vivo application of quantitative mapping of total tissue membrane potential, *ΔΨ*_*T*_.

## Introduction

Mitochondria produce approximately 90% of cellular adenosine triphosphate (ATP) through oxidative phosphorylation [1]. The electron transport chain (ETC) of the mitochondrion is ultimately responsible for converting the foods we eat into electrical and chemical energy gradients by pumping protons across the inner membrane in the mitochondrial intermembrane space. The energy stored in the electric field, referred to as mitochondrial membrane potential (ΔΨ_m_), is then used to power the conversion of ADP to ATP. In a typical cell, the ΔΨ_m_ remains constant with time and is about -140 mV [2]. Table 1 lists ΔΨ_m_ for mitochondria of different cell types.

**Table 1:** Measured values of mitochondrial membrane potential (milliVolts).

Authors	Preparation	Tracer Method	Δψ_m_ (mV)	Δψ_T_ (mV)
This work	in vivo swine heart	^18^F-TPP^+^ PET	_____	-129
Kauppinen, R.[3]	perfused rat hearts	^3^H-TPMP^+^	-125	
Rottenburg, H.[4]	rat liver	^3^H-TPP^+^	-150	
LaNoue, et al.[5]	isolated brown adipocytes	^3^H-TPP^+^	-116	
Wan, et al.[6]	perfused rat hearts	^3^H-TPP+	-145	
Gurm, et al.[7]	in vivo swine heart	^18^F-TPP^+^	-91	
Gerencser et al. [8]	cultured neurons (resting)	Fluorescent probe	-139	
Kamo et al.[9]	isolated mitochondria (state 4)	TPP^+^ electrode	-180	

Column 1 indicates the author(s) of each study. Column 2 indicates the conditions of measurement, e.g. isolated cells. Column 3 indicates the methodology used in the measurement. Column 4 list the value of ΔΨ_m_. Column 5 lists the value of ΔΨ_T_

If ΔΨ_m_ remains within the physiological range, a small amount of reactive oxygen species (ROS) is produced. However, in mitochondrial dysfunction, ΔΨ_m_ falls outside the normal range, with concomitant increase in ROS release, and impairment of ATP production [10]. And because mitochondria are the most important source of energy and ROS in the cell, mitochondrial dysfunction is at the core of many diseases, including myopathies [11], diabetes [12], degenerative diseases [13], inflammation [14], cancer [15], and cardiac arrhythmias [16, 17].

Despite continuing scientific interest in voltage sensitive probes, a noninvasive method for measuring ΔΨ_m_ in living animals does not currently exist. The basic physiological studies conducted several decades ago are highly relevant but not always mentioned: Historically, fluorescent dyes [18] and lipophilic cationic tracers have been developed for quantitative assay of ΔΨ_m_ in isolated mitochondria [4], cells [19], and isolated heart preparations [6]. Electrodes sensitive to tetraphenylphosphonium (TPP) have also been developed and used to evaluate ΔΨ_m_ in isolated mitochondrial fractions [9, 20]. [^14^C or ^3^H]-labeled lipophilic cations were used to study the electrical properties of membranes and mitochondria long before modern imaging methods were imagined [3, 21–23]. More recently, Logan et al. [24] reported a "click" method for in vivo measurement of ΔΨ in the cells of mouse hearts, but their method requires the excision of the heart and hence is not suitable for translation to human studies.

The work cited above established the use of ^3^H-TPP^+^ as a reference tracer for measuring mitochondrial membrane potential (ΔΨ_m_). Investigators have shown that ^3^H-TPP^+^ distributes slowly in accord with the electrochemical gradient [4, 22]. Min et al suggested that TPP might be an important imaging agent [25].By replacing the tritium label with ^18^F[26], it is possible to adapt the methodology to PET, making it feasible to extend these measurements to intact animals and to human studies. In this paper, we report work that adapts the methods used in the earlier bench top measurements to in vivo imaging using positron emission tomography (PET/CT) to measure and map total membrane potential (ΔΨ_T_) We define ΔΨ_T_ as the sum of *ΔΨ*_*m*_ and cellular (*ΔΨ*_*c*_) electrical potentials. The time scale of our measurements are tens of minutes and thus ΔΨ_c_ is represented by its time average. ΔΨ_T_ is chosen as a practical surrogate for ΔΨ_m_, keeping in mind that in most situations ΔΨ_m_ ≈ 10*ΔΨ_c_ and thus ΔΨ_T_ ≈ ΔΨ_m_. In our methodology a cationic lipophilic tracer TPP^+^, labeled with ^18^F, is used to quantitatively map myocardial ΔΨ_T._^18^F-TPP^+^ was initially developed as a myocardial flow imaging agent under the trade name BFPET [26] However, ^18^F-TPP^+^ enters the tissue with a low first-pass extraction fraction and does not respond to pharmacological challenge with a stressor and hence cannot be considered a flow tracer [7]. Nonetheless, its electrochemical properties make it a tracer of interest for quantitative imaging of ΔΨ_T_.

Previous work, attempting to detect changes in concentration due to alteration of the ΔΨ_m_ used tracers such as ^99m^Tc-sestamibi [27], tetraphenyl phosphonium[25], (18F-fluoropentyl) triphenylphosphonium[28], and ^18^F-fluorobenzyl triphenyl phosphonium [29] [30], with semi-quantitative endpoints such as SUV [31]. The results of these studies are empirical and descriptive. In the sense that a binary decision threshold is sought; the electrical properties of transmembrane kinetics are not exploited for quantitative purposes. Changes indicative of graded mitochondrial dysfunction cannot be detected. Gurm et al reported the first attempt to quantitatively measure ΔΨ_m_ with PET and ^18^F-TPP^+^ [7]. Their analysis simply applied the Nernst equation to the PET and plasma concentrations measured 30 minutes after bolus injection of ^18^F-TPP^+^. But terminating the study at 30 minutes was arbitrary and did not consider that the plasma level falls monotonically for at least 120 minutes, meaning that had they used the data at, say, 45 minutes after injection, they would have obtained a different result. Thus, their assumption that tracer was in steady state 30 minutes after bolus injection is incorrect and leads to biased results. In addition, their analysis did not consider the effect of tracer in the extracellular space. Because of these errors, their method violates basic tracer kinetic principles and their results significantly underestimate ΔΨ_m_ and are not in good agreement with work from in vitro studies (Table 1).

### General design of the studies

This investigation provides an initial assessment of a method for quantitative mapping of ΔΨ_T_. Because the mammalian heart has the highest concentration of mitochondria, we chose myocardial imaging as the first application to facilitate optimization of the scanning conditions. The subjects of our study were domestic swine (Sus domesticus) imaged in one of two conditions: healthy controls or pigs with chronic injury to the left anterior descending arterial (LAD) territory. We used bolus injection of ^18^F-TPP^+^ in control and injured pigs to measure the kinetics of TPP^+^ for at least two hours, determining the volume of distribution by a regression model.

## Methods

### A new method for quantification of membrane potential

**Fig 1:**
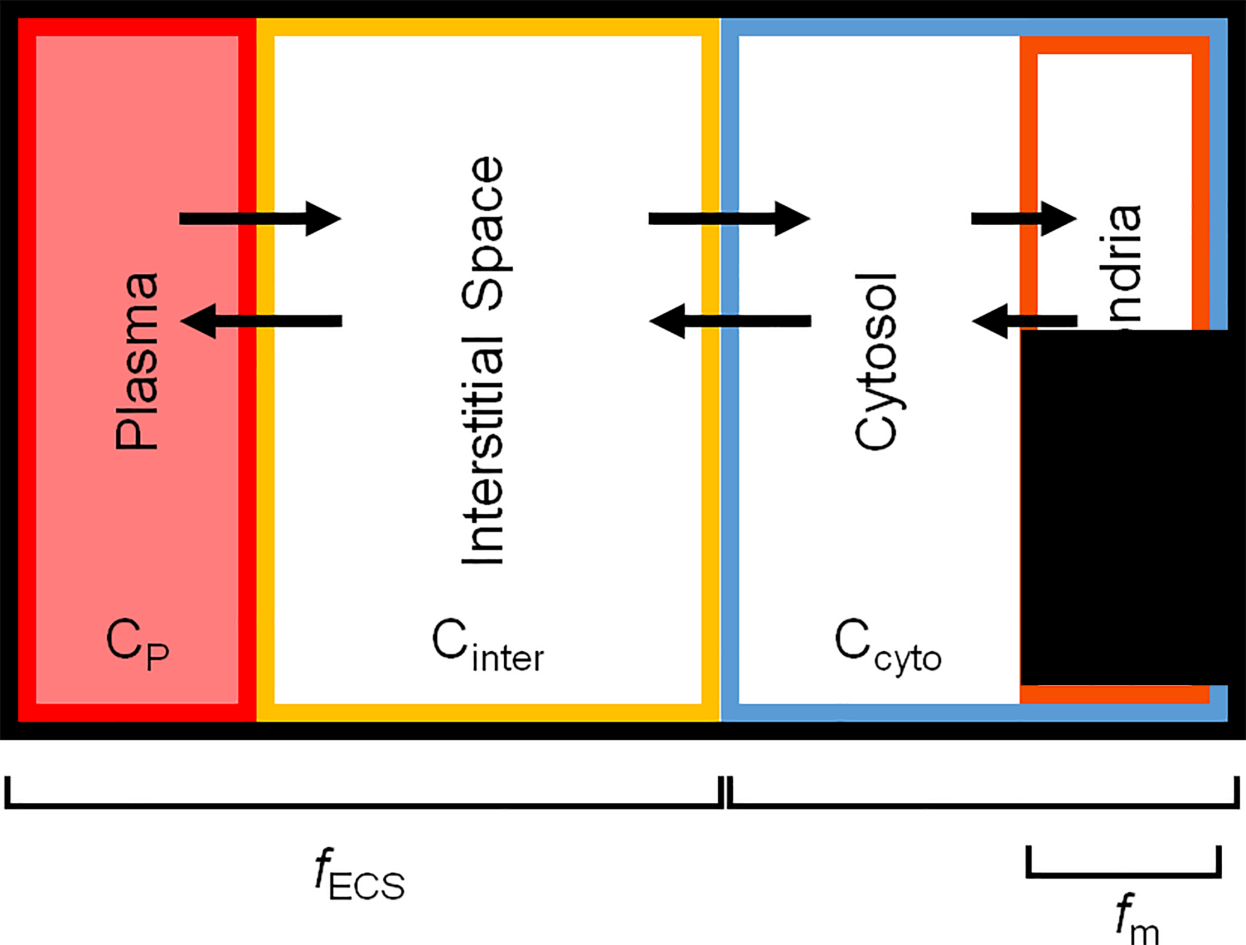
Volume of distribution model for^18^F-TPP^+^ in a PET image voxel. The outer black line represents the voxel boundary. C_p_, C_inter_, C_cyto_, and C_mito_ represent the concentrations of the plasma, interstitial space, cytosol, and mitochondria respectively. The arrows represent ^18^F-TPP^+^ transport between the different compartments. *f*_ECS_ represents the voxel volume fraction occupied by ECS and *f*_mito_ represents the cellular volume fraction occupied by mitochondria.

We partition the tissue distribution of ^18^F-TPP^+^ into several components: ECS, consisting of interstitial space and plasma, mitochondria (mito) and cytosolic (cyto) volume fractions (Fig 1). The Nernst equation [32, 33] equates transmembrane electric potential to the ratio of ion concentrations on either side of the membrane. Thus, the Nernst equation allows us to derive an equation relating the PET measurements of ^18^F-TPP^+^ concentration, to Δ*Ψ*_*T*_, ECS fraction (*f*_*ECS*_), mitochondrial volume fraction (*f*_*mito*_) and the electric potential across the cellular membrane, ΔΨ_c_. At steady state, the concentration of ^18^F-TPP^+^ in a PET voxel can be written as

$${\bar{C}}_{PET}={(1-f}_{ECS})(f_{mito}∙{\bar{C}}_{mito}+(1-f_{mito})∙{\bar{C}}_{cyto})+f_{ECS}∙{\bar{C}}_{ECS}$$ (1)

where, ${\bar{C}}_{mito}$, ${\bar{C}}_{cyto}$ and ${\bar{C}}_{ECS}$ are the steady state concentrations of TPP^+^ in the mitochondria, cytosol and ECS, respectively. At steady state plasma and ECS are equal and ${\bar{C}}_{mito}$ and ${\bar{C}}_{cyto}$ are related through the Nernst equations:

$$\frac{{\bar{C}}_{cyto}}{{\bar{C}}_{ESC}}=e^{-\beta {\Delta \Psi }_{c}}\;\mathrm{and}\;\frac{{\bar{C}}_{mito}}{{\bar{C}}_{cyto}}=e^{-\beta {\Delta \Psi }_{m}}$$ (2)

We divide Eq 1 by ${\bar{C}}_{p}$ and use Eq 2 to express the tracer volume of distribution, V_T_, as

$$V_{T}=\frac{{\bar{C}}_{PET}}{{\bar{C}}_{p}}={(1-f}_{ECS})(f_{mito}∙e^{-\beta \Delta \Psi_{T}}+(1-f_{mito})∙e^{-\beta \Delta \Psi_{c}})+f_{ECS},$$ (3)

where $\beta =\frac{zF}{RT}$ is a ratio of known physical parameters: F denotes Faraday's constant, z is the valence, R is the universal gas constant and T is the temperature in degrees Kelvin. In our calculations z = 1, F = 96485.3 [Coulombs per mole], R = 8.314472, and T = 310.2 [degrees Kelvin]. $\frac{{\bar{C}}_{PET}}{{\bar{C}}_{p}}$ is the steady-state ratio of the tissue to plasma concentrations. This equation predicts that V_T_, a kinetically determined tracer quantity, is equal to the steady state concentration ratio $\frac{{\bar{C}}_{T}}{{\bar{C}}_{p}}$. Thus, when the tracer concentrations in tissue and plasma are time-invariant, V_T_ is, by definition, the tissue-to-plasma concentration ratio; whereas, after bolus injection the tissue-to-plasma concentration ratio varies with time and V_T_ must be determined kinetically.

Unlike tracers whose distribution is governed by passive transport, ^18^F-TPP^+^ will have a very high (>>1) volume of distribution when the membrane potential is in the normal range. A reasonable approximation to the volume of distribution is given by

$$V_{T}=\frac{{\bar{C}}_{T}}{{\bar{C}}_{p}}\approx (1-f_{ECS})∙f_{mito}∙e^{-\beta ∙{\Delta \Psi }_{T}}$$ (4)

**Fig 2:**
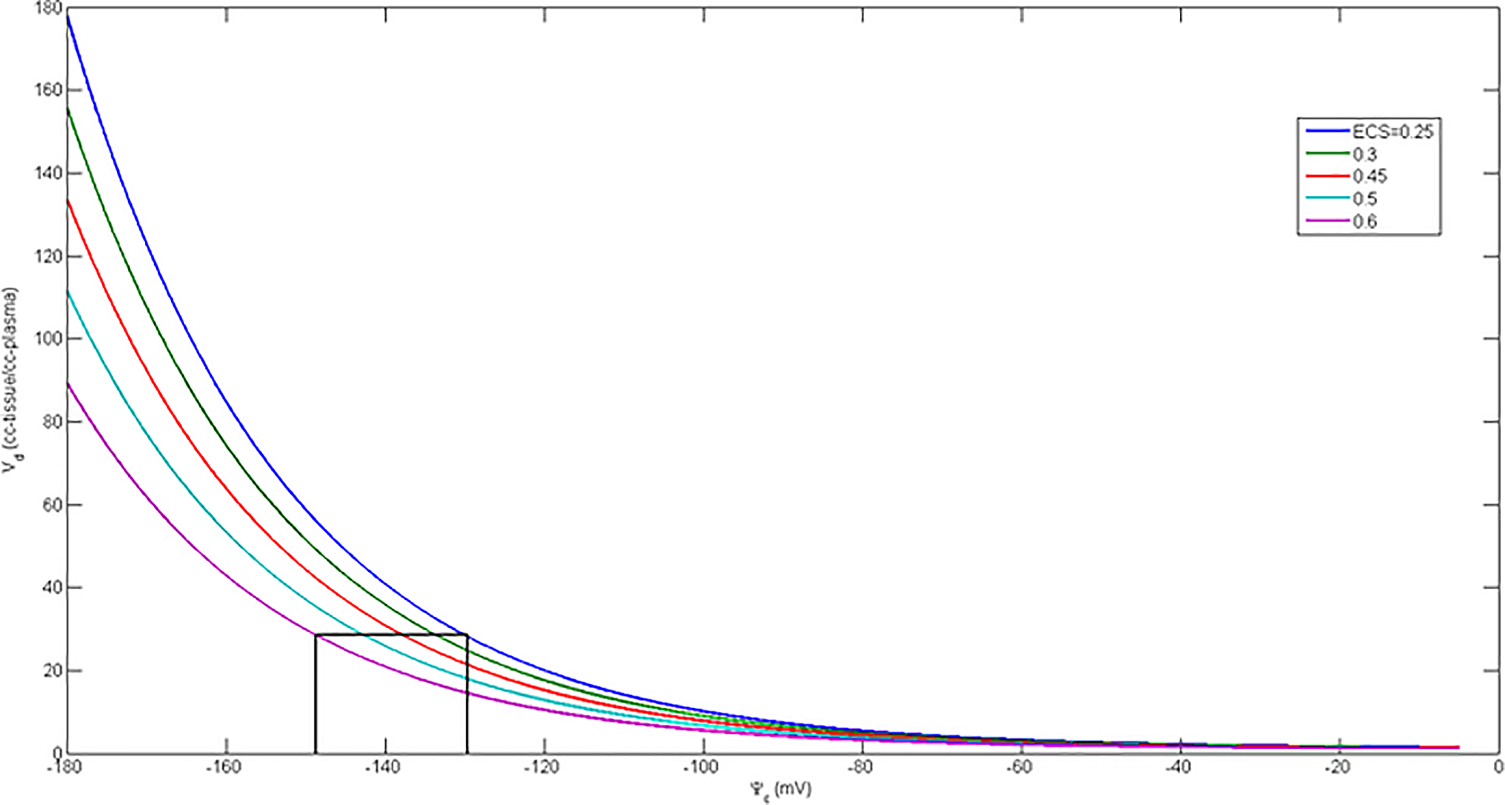
Dependence of *V*_*T*_ on mitochondrial membrane potential and fractional ECS computed with Eq 3. *ΔΨ*_*T*_ is more negative inside membranes. Black lines indicate variation (systematic error) in *ΔΨ*_*T*_ range at constant volume of distribution due to neglecting fractional ECS volume.

Barth et al. [34] have studied 10 different mammalian species, including pigs and man, finding that the myocardial *f*_*mito*_ is a specific and constant value for any particular species. Therefore, V_T_ is sensitive to two independent variables; Δ*Ψ*_*T*_ and *f*_*ECS*_. The fundamental mathematical relationships expressed in Eqs 1, 2 and 3 are depicted in Fig 2, illustrating that the systematic error in Δ*Ψ*_*T*_ due to neglecting *f*_*ECS*_ for normal values of *V*_*T*_ is about 20 mV. But note that Fig 2 also shows that at low membrane potential, the effect of the ECS is negligible, meaning that knowledge of *f*_*ECS*_ is most important for detecting normal versus mildly dysfunctional mitochondria.

### Study cohort

^18^F-TPP^+^ scans were performed on eight Yorkshire swine (Pigs were all American Yorkshire male ordered from Animal Bioware^TM^ Series II Sofware Suite, vendor: Tufts). There were 5 animals without tissue injury, referred to as control pigs, and 3 animals with a left anterior descending artery (LAD) infarction, referred to as injury pigs. Pigs shared a room with other animals of the same species, individually housed in their own cage, free to turn and make normal movements and postural adjustments. The animals were given play toys to enrich their environment and fed adequately, with ready access to water, to ensure normal growth. An acclimation period greater than 4 days was observed upon the animal’s arrival before conducting the first imaging procedure.

Animals used in this study were housed and maintained under the supervision of the Massachusetts General Hospital Animal Care and Use Committee and our study was conducted under a protocol approved by the Institutional Animal Care and Use Committee of the Massachusetts General Hospital.

### Anesthesia

Following a 12 hour fast, pigs were sedated with 4.4 mg/kg Telazol. Anesthesia was induced with isoflurane 5% and maintained with 1.5% isoflurane. During anesthesia, the animals were mechanically ventilated. Vital signs and depth of anesthesia were assessed once every 5 minutes and this assessment was documented every 15 minutes.

### Tissue injury

Percutaneous central access was achieved via the Seldinger technique [35] under anesthesia. Femoral artery access was obtained and a guide wire was advanced into the LAD coronary artery. A balloon catheter was fed over the guidewire, placed in the mid LAD, and inflated to 6–8 atm for 80 minutes. An infarct was confirmed by the appearance of large ST elevation on ECG. At the end of the surgical procedure, subcutaneous Carprofen was administered and the animal was returned to housing for recuperation. After the immediate postoperative period, the animals were observed at least twice daily by study staff. Post-procedural Carprofen was administered orally for 3 days at a dose of 150 mg/day and then as needed if stiffness or swelling continued after that point. In order to prevent arrhythmias, 200mg Amiodarone and 50 mg Atenolol were also given orally every day during 2 weeks.

During the study, animal health and well-being, as well as the adequacy of anesthesia, were monitored by checking respiration rate, ECG, blood gas, corneal or palpebral reflex, blood pressure, heart rate, pulse oximetry.

### Blood sampling

Radiotracer injection, iodinated contrast injection, gadolinium injection, and venous blood sampling were performed through femoral vein catheters. An arteriovenous shunt was placed in the left femoral artery for arterial blood sampling. After tracer injection, arterial blood samples were obtained every 10 seconds for the first 3 minutes, then at 1 minute intervals for 5 minutes, and at increasing intervals until 120 minutes post injection. Venous blood samples were obtained at 5, 10, 15, 30, 60 and 90 minutes after tracer injection. All blood samples were centrifuged to determine plasma and red cell concentration histories.

### Scanning

Eight scans, 5 control and 3 injury pigs, were performed using a Siemens Biograph 64 PET/CT. Following administration of 185 MBq of 18F-TPP^+^ as a single intravenous bolus, scanning was performed over 120 minutes in list mode. CT angiography was performed for anatomic reference. List mode data were framed as a dynamic series of 12x3, 9x5, 7x10, 15x30 second frames. PET/CT data were reconstructed using a filtered back projection algorithm with CT-based attenuation correction to yield a radioactivity concentration map in units of Bq/cc with 83 slices and a voxel size of 2.14x2.14x3 mm^3^. *f*_*ECS*_ was measured in 3 injury pigs with CT scanning using a bolus plus infusion iodinated contrast protocol [36].

### Data analysis

Dynamic PET data were analyzed using a Logan regression method [37] to produce quantitative parametric images of the V_T_ of ^18^F-TPP^+^. Images of V_T_ were reoriented into the short axis projection and cropped so that the cardiac chambers occupied nearly all of the image space. Eq 3 was used to convert the parametric maps of V_T_ to maps of ΔΨ_T_, assuming ΔΨ_c_ = -15 mV [7] and *f*_mito_ = 0.26 [34]. Segmental values are reported as a grand mean ΔΨ_T_ and its standard error of the mean (SEM). No background subtraction or thresholding was applied to the parametric images of *V*_*T*_.

## Results

### In vivo mapping of ΔΨ_T_

Fig 2 was computed using Eq 3 to show the predicted behavior of mitochondrial membrane potential as a function of total volume of distribution, and size of the extracellular space. ΔΨc was assumed to be -15 mV for these calculations. Fig 2 shows a nearly exponential behavior and that *ΔΨ*_*T*_ is nearly independent of *f*_*ECS*_ when membrane potential drops below about -100 mV.

**Fig 3:**
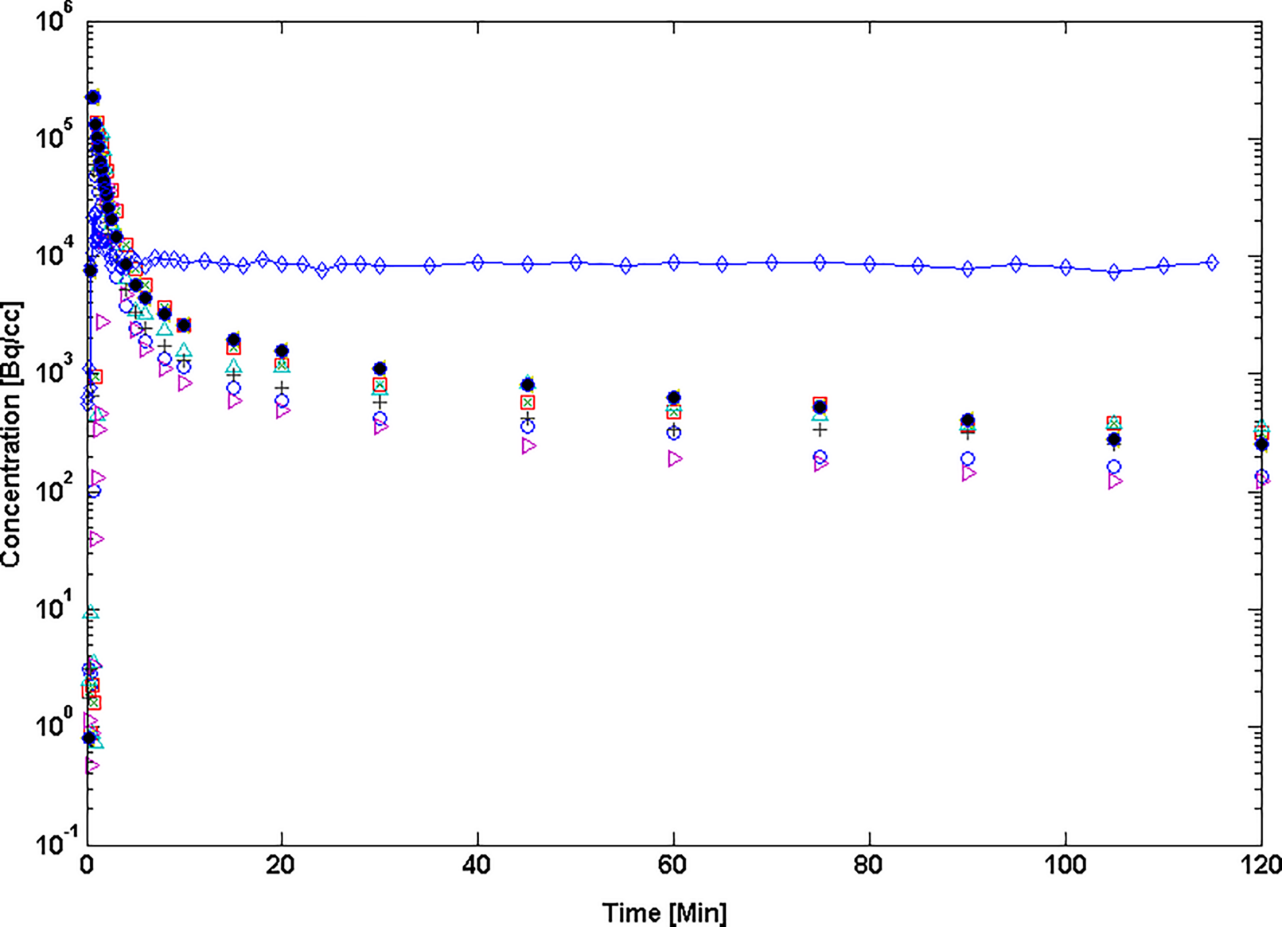
Variation of plasma and tissue concentration following intravenous bolus injection of ^18^F-TPP^+^. Plasma concentration decreases monotonically over the first two hours; whereas, myocardial concentration is nearly constant.

**Fig 4:**
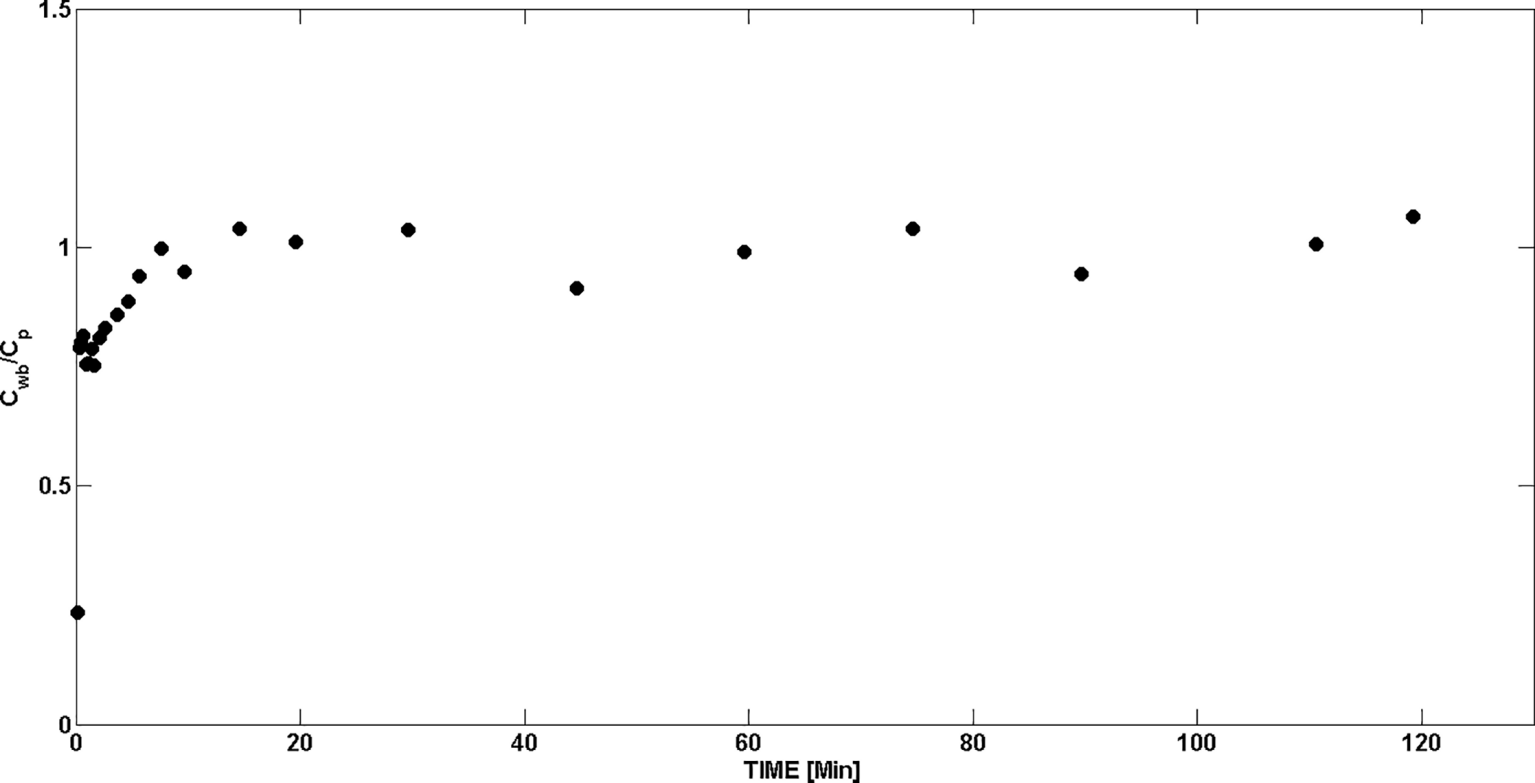
Whole blood-to-plasma concentration ratio measured as a function of time after bolus injection of ^18^F-TPP^+^. After about 15 minutes, whole blood and plasma concentrations equilibrate with equal concentration.

As shown in Fig 3, after bolus injection, the plasma concentration history decreased rapidly during the "equilibration" phase and slowly thereafter; whereas, TPP demonstrated an extended myocardial residence time characterized by nearly constant or slowly decreasing tissue concentration. The concentration ratio for whole blood versus plasma became constant, with a mean value of 0.98±0.02 (SEM), about 15 minutes after injection of TPP^+^ (Fig 4). Venous and arterial samples obtained later than 10 minutes after injection of TPP^+^ were in good agreement.

^18^F-TPP^+^ concentration was highest in heart and liver, reaching a plateau in normal myocardium after about 10 minutes and declining very slowly thereafter. The uptake of ^18^F-TPP^+^ in scar was variable, reflecting the variation in degree and extent of injury. Tissue concentration in the injured area peaked about 10 minutes after injection, followed by a slow biphasic clearance, with the plateau level about 40% as high as in the normal myocardium. In normal left ventricular myocardium, average *f*_*ECS*_ = 0.20 and varied less than 10%. Local values of *f*_*ECS*_ could not be obtained in the tissue injury, due to poor signal-to-noise ratio in the CT-studies and average values were used in computation of *ΔΨ*_*T*_.

**Fig 5:**
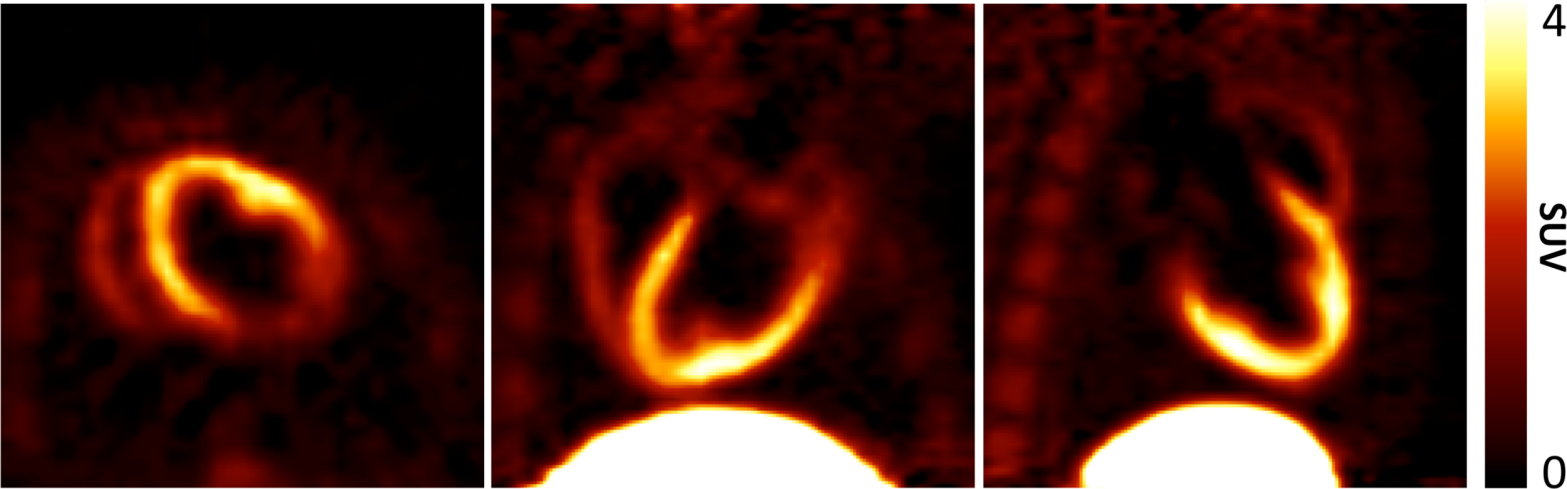
Typical ^18^F-TPP^+^ SUV image, integrated from 60–120 minutes after IV bolus injection. SUV is highest in liver, followed by LV myocardium, with lower activity in the visible in bone marrow.

TPP^+^ is avidly taken up by normal LV myocardium, as shown in Fig 5. The distribution of ^18^F-TPP^+^ is shown as SUV integrated from 60–120 min post tracer injection in the three oblique projections obtained directly from the PET image volume. No further processing was done to these images.

**Fig 6:**
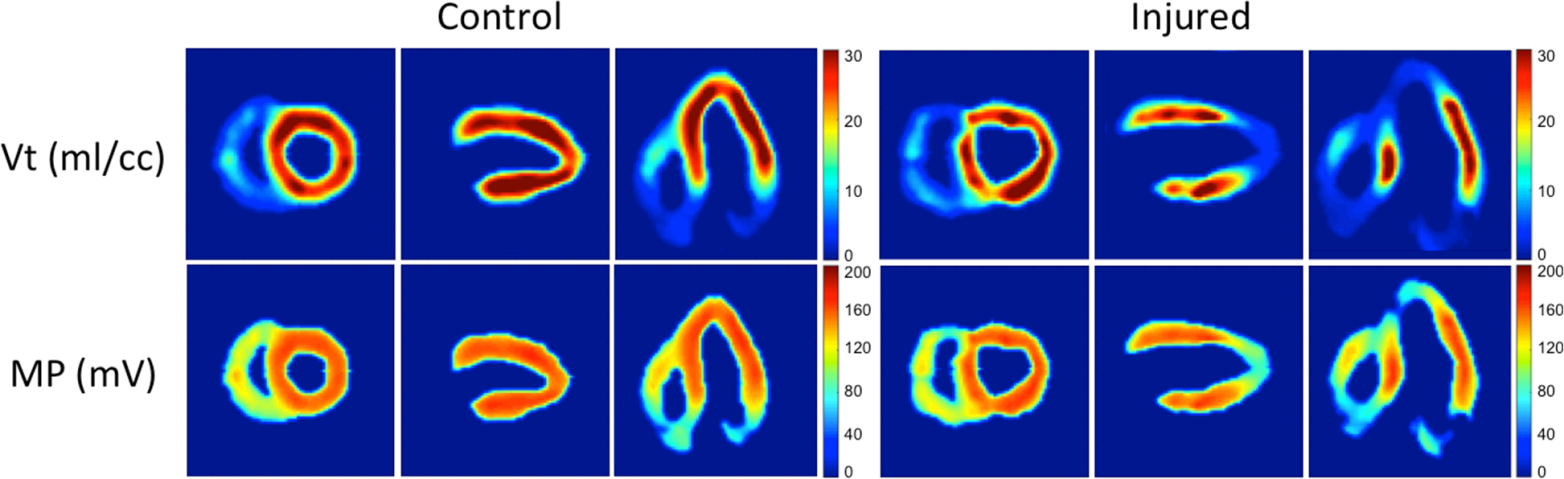
Parametric images of V_T_ and ΔΨ_T_. Each panel of three x two images shows short axis, vertical and horizontal slices. Images of a representative control pig are shown in the left panel. Images of a representative scar pig are shown in the right panel. The top row of each panel depicts the TPP^+^ volume of distribution and bottom row the membrane potential.

Representative parametric images of *V*_*T*_ and *ΔΨ*_*T*_ derived from kinetic data obtained from a control pig and an injury pig were reoriented into the standard cardiac coordinate system and presented in Fig 6. Images of *V*_*T*_ have units of cc-tissue/g-plasma; whereas, images of *ΔΨ*_*T*_ are in units of negative millivolts (-mV). The apparent lower volume of distribution in right ventricle and atria is artifactual, due to the thinner walls of those structures in combination with the effects of finite spatial resolution and cardiac motion blurring.

**Fig 7:**
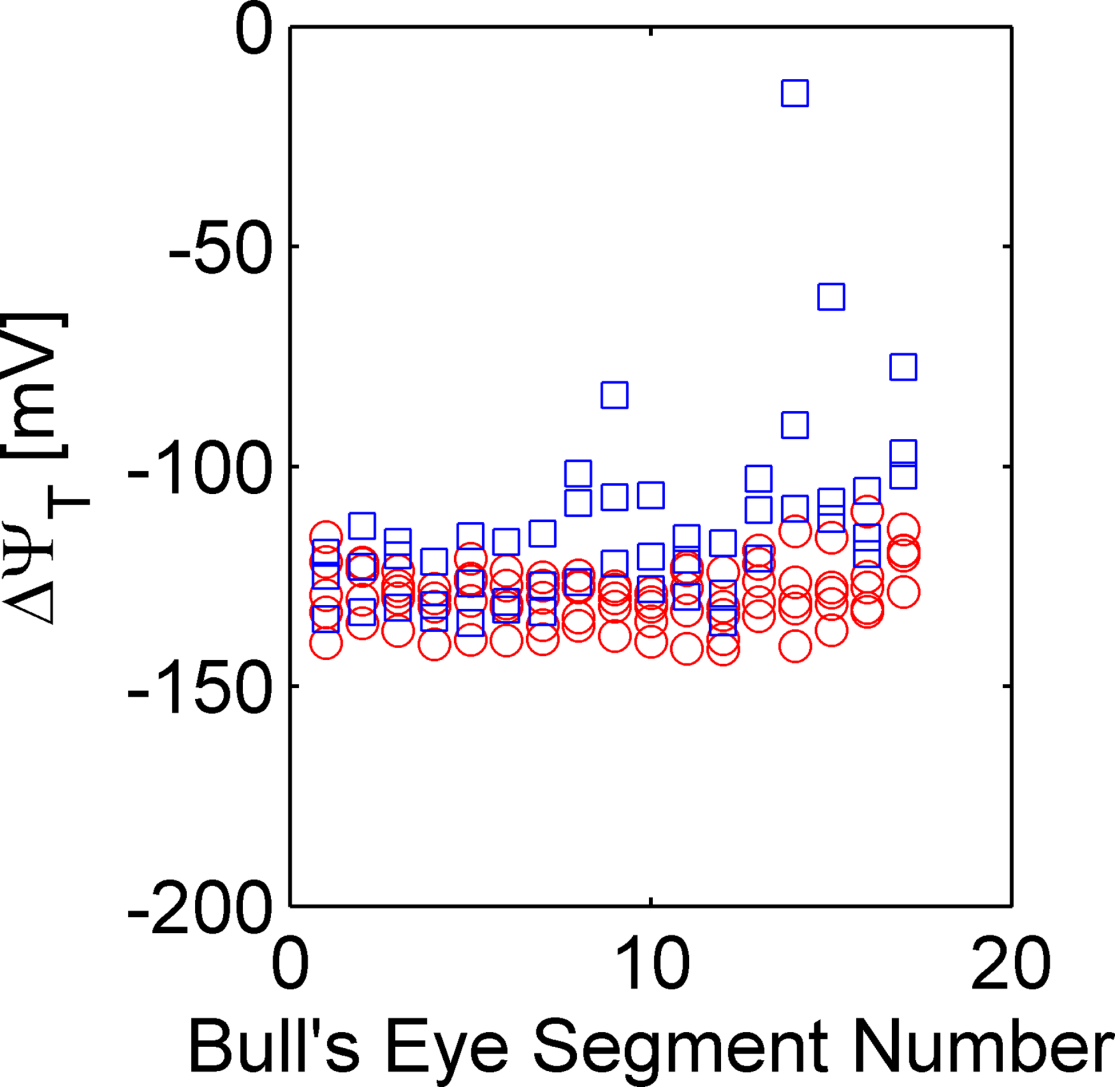
Comparison of *ΔΨ*_*T*_ in 17 "bull’s eye" segments Results shown for five control (blue squares) and three pigs with injury to the LAD territory (red circles).

Segmental *ΔΨ*_*T*_ is tightly grouped for the five control pigs with a grand mean ± SEM over 17 segments of -129.4±1.4 mV (Fig 7). Values of *ΔΨ*_*T*_ are lower in injured segments, particularly in the apical-septal segments corresponding to the injury in the territory of the LAD artery.

## Discussion

In this paper, we introduce the concept of quantitative mapping of *ΔΨ*_*T*_ for monitoring mitochondrial status. Our development emphasizes measurement of the total membrane potential, *ΔΨ*_*T*_, while making clear that *ΔΨ*_*T*_ is a proxy for and tightly correlated to *ΔΨ*_*m*_. Direct measurements of *ΔΨ*_*m*_ require a separate measurement of the cellular membrane potential that is currently not possible in vivo. Just as in the early studies conducted with ^3^H-TPP^+^, our method relies on measuring the total concentration of a lipophilic cationic tracer which is then analyzed by using a compartment model of the tissue and the steady state formulation of the Nernst equation, the same equation that is fundamental to electrochemistry [38] and cardiac electrophysiology [39, 40].

The relation of tracer concentration, which varies, and the physiological steady state can seem confusing. In the physiological state all the biological concentrations, transport rates and electropotentials are assumed to be fixed; whereas, the tracer concentrations evolve over time during the experiment (Fig 3). This complicates the application of the Nernst equation, whose use assumes the concentrations determining the membrane potential are invariant with time. We addressed that issue by using a kinetic model with extracellular and intracellular pools measured with dynamic PET to estimate the total volume of tracer distribution, a time invariant quantity characteristic of the tracer and the animal under study. We explicitly considered the effect of the electric field across the inner membrane of the mitochondrion on the kinetics of TPP^+^ by using a kinetic model to express the relation between the total volume of distribution of the tracer and the electrical properties of the membrane. The structure of this model is identical to that used to analyze the classic ^3^H-TPP^+^ experiments conducted many years ago.

While qualitative imaging may serve many purposes, the quantitative aspects of our method may provide additional important information about mitochondrial status not available from visual inspection of images, true because mitochondrial dysfunction may, in some tissues, be associated with uniformly reduced *ΔΨ*_*m*_ and such conditions cannot be detected by qualitative imaging methods.

Another potentially important result of this work is the more analytic understanding of the factors affecting the distribution of TPP^+^, in particular, and voltage sensing tracers in general. We used basic principles of physics and physiology to show how the volume of distribution of lipophilic cations depends on the volume fractions of the tissue occupied by the ECSand the mitochondria as well as on the magnitude of ΔΨ_T._ V_T_ is approximately equal to $(1-f_{ECS})∙f_{mito}∙e^{-{\Delta \Psi }_{T}}$. Keeping in mind that for normal tissue the mitochondrial membrane potential is about -140 mV [2] for all mitochondria regardless of tissue type; whereas, the fractional mitochondrial volume varies by more than a factor 10, Eqs 3 and 4 imply that the intensity of the V_T_ image will reflect the mitochondrial volume fraction of the tissue, thereby explaining the intensity variations depicted in Fig 5. Eqs 3 and 4 also make clear that the size of the ECS is an important factor when interpreting TPP^+^ images because this quantity may vary with age and disease. Fig 2 shows the model-prediction for the volume of distribution when we fix the mitochondrial volume fraction while varying the membrane potential and the fractional volume of the ECS. This result shows that increases in the size of the ECShave decreasing effect on the total volume of distribution as the membrane potential decreases and becomes depolarized. But if the goal is to detect more subtle changes from normal ΔΨ_T_ it is important to include the effect of variation in ECS. Failure to account for changes in ECS may lead to unexplained variability in the qualitative and quantitative assessments of voltage-sensing tracer distribution.

*ΔΨ*_*m*_ is sustained by the electron transport chain of the mitochondria, by which a balance is struck between protons pumped across the inner mitochondrial membrane and those pumped back to power the synthesis of ATP. *ΔΨ*_*m*_ is also affected by changes in the level of ROS and various mitochondrial ion channels [41]. Interestingly, increased ROS levels and modulation of mitochondrial ion channel function are seen early in numerous pathologies. Thus, the ability to quantitatively map *ΔΨ*_*T*_ may be useful for diagnosing or managing a number of such conditions. For example, reduction in *ΔΨ*_*m*_ has been implicated as a mechanism underlying ventricular arrhythmogenesis [42] and so might be used to improve the detection of arrhythmogenic foci.

We noted in Introduction that there are currently no methods for measuring *ΔΨ*_*m*_ in animal or human. This means that there is no independent method against which we can compare our PET methods. In this regard, it is important to emphasize that our noninvasive PET method is strongly related to the highly invasive and validated bench-top methods which preceded it. All prior methods employing in vitro application of cationic tracers were based on the Nernst equation to relate steady state concentrations and membrane potential in a compartment model of the mitochondrion, cell or tissue. Similarities to PET can be seen with ^3^H-TPP^+^ studies conducted in populations of cells and isolated mitochondria, where concentrations of TPP^+^ in the medium are related to the concentration in ensembles of cells or mitochondria by the Nernst equation. Measurements with TPP^+^ electrodes are also based on the same principles [9]. The similarity is most apparent in the work of Wan et al. [6] who studied *ΔΨ*_*m*_ in isolated rat hearts by using the arterio-venous difference in concentration of ^3^H-TPP^+^ to infer the tissue-to-perfusate concentration ratio. *In essence*, *measuring the evolution of* ^*18*^*F-TPP*^*+*^ *concentration in a PET ROI is completely analogous to measurements with* ^*3*^*H-TPP+ in the isolated perfused rat heart studies of Wan et al*. [6]. Hence, the PET studies are the natural extension of the classical bench top reference methods.

The fact that our PET measurements of *ΔΨ*_*T*_ are in accord with measurements in isolated rat hearts is an important observation, especially since direct validation of our method is impossible with existing methodologies. As illustrated in Table 1, the PET method yields results that are close to those found by the majority of prior studies, thereby providing additional support of our method.

With high specific activity, the mass of ^18^F-TPP^+^ is 10^−10^ to 10^−7^ lower than the intracellular potassium concentration, meaning its effect on membrane potential is negligible. However, it is worth mentioning that prior studies with ^3^H-TPP^+^ have sometimes made corrections for non-specific binding of TPP^+^ [4] but the literature is not concordant on the necessity of correction [4, 9, 43–45]. Furthermore, previous bench top studies used very low specific activity TPP^+^, complicating an evaluation of its necessity in tracer measurements. Nevertheless, similar corrections could be applied to the PET analyses, but were found to be unnecessary to establish initial validity.

Close inspection of Fig 5 shows there is a high value of *V*_*T*_ in normal myocardium, with mitochondrial concentrations nearly 30 times the plasma level. At secular equilibrium, the inward and outward fluxes across the mitochondrial membrane have to balance, meaning that outward clearance must be about 30 times lower than the inward rate, thereby explaining the slow clearance observed experimentally after bolus injection of ^18^F-TPP^+^. Fig 5 also shows that the volume of distribution in injured myocardium is much lower, with tissue concentrations less than 10 times the plasma concentration. Our kinetic model shows that *V*_*T*_ depends linearly on *f*_*ECS*_ and exponentially on *ΔΨ*_*T*_. Accordingly, we have converted *V*_*T*_ to an estimate of *ΔΨ*_*T*_ by accounting for the effects of *f*_*ECS*_, a quantity known to vary in age and disease [46–49]. Thus, we have shown it will be necessary to account for changes in extracellular volume fraction in clinical studies to obtain the full benefit of such studies.

A marked difference in contrast between *V*_*T*_ and *ΔΨ*_*T*_ was observed between normal and injured tissue (Fig 5). Both *V*_*T*_ and *ΔΨ*_*T*_ are quantitative measures emphasizing different aspects of ^18^F-TPP^+^ distribution. On one hand, *V*_*T*_ is the total volume of ^18^F-TPP^+^ distribution, including effects due to *ΔΨ*_*T*_, *f*_*ECS*_, and *f*_*mito*_. Therefore, mitochondrial density (*f*_*mito*_) will affect the relative uptake of a voltage-sensing tracer. This finding is interesting given that different pathologies are associated with reduced mitochondrial density. For example, a reduction in mitochondrial density is seen in the skeletal muscles of patients with chronic obstructive pulmonary disease [50]. In these conditions, *V*_*T*_ measurements would provide an overall quantitative measure of mitochondrial status in tissue. On the other hand, *ΔΨ*_*T*_ focuses predominantly on the electrochemical conditions that prevail at the inner mitochondrial membrane. In the large tissue injury, we see that there is an area of profound reduction in *ΔΨ*_*T*_, approaching total depolarization. Other parts of the injured region show lesser depolarization in the range of -60 mV, but still very different than *ΔΨ*_*T*_ in normal myocardium. This can also be appreciated from the data in Fig 7, where the blue circles indicate major reductions in the average *ΔΨ*_*T*_ for bull’s eye segment 8, 9, 14, 15 and 17. We can also see the variability of tissue injury expressed in Fig 7, which demonstrate a patchy nature to those injuries that might be better appreciated by direct examination of the parametric images.

As also shown in Fig 7, pigs in the control group exhibited segmental values of *ΔΨ*_*T*_ that averaged about -129.4 ± 1.4 mV (SEM). The tight grouping of *ΔΨ*_*T*_ measurements over all normal segments and pigs is remarkable. The kinetic approach, used in this study, requires a long measurement period for accurate estimation of the total volume of distribution. A protocol using primed constant infusion may be preferred for human investigation in order to restrict actual scan time to the equilibrium period, 95–120 min.

In this work we reported the results of kinetic analysis using the Logan graphical method, which is known to underestimate *V*_*T*_ with increasing statistical noise in the measurements[51]. We mitigated the effect of statistical noise by averaging *ΔΨ*_*T*_ maps for 17 polar segments but the reader should be aware that the spatial averaging also causes some underestimation of *ΔΨ*_*T*_. We also formed parametric images using the reGP method of Zhou et al [52] and the total least squares approach of Varga and Szabo,[53] but these methods yielded noisy parametric images; overall, they were not an improvement over the Logan plot.

## Conclusions

This study is the first to demonstrate the feasibility of quantitative in vivo mapping of total membrane potential, *ΔΨ*_*T*_, a proxy of *ΔΨ*_*m*_. In vivo measurements of *ΔΨ*_*T*_ obtained with our new method yielded values remarkably constant within and across the hearts of domestic swine that are comparable to results from in vitro bench top experiments. We have derived a theory explaining, for the first time, the major factors affecting the transport and residence time of lipophilic cations in tissue, including *ΔΨ*_*T*_ and *f*_*ECS*_. The fact that we can measure ΔΨ_T_ in mV suggests that it may be possible to compare individual's studies with normative data. Given the critical role of mitochondrial function in numerous pathologies, the potential applications of this new imaging method are immense. This novel technique could eventually be proved useful in numerous clinical and research scenarios.
