# Correction: Quantitative *in vivo* mapping of myocardial mitochondrial membrane potential

**DOI:** 10.1371/journal.pone.0192876

**Published:** 2018-02-08

**Authors:** The PLOS ONE Staff

Fig 1 is incorrect. Please see the correct Fig 1 here. The publisher apologizes for this error.

**Fig 1:**
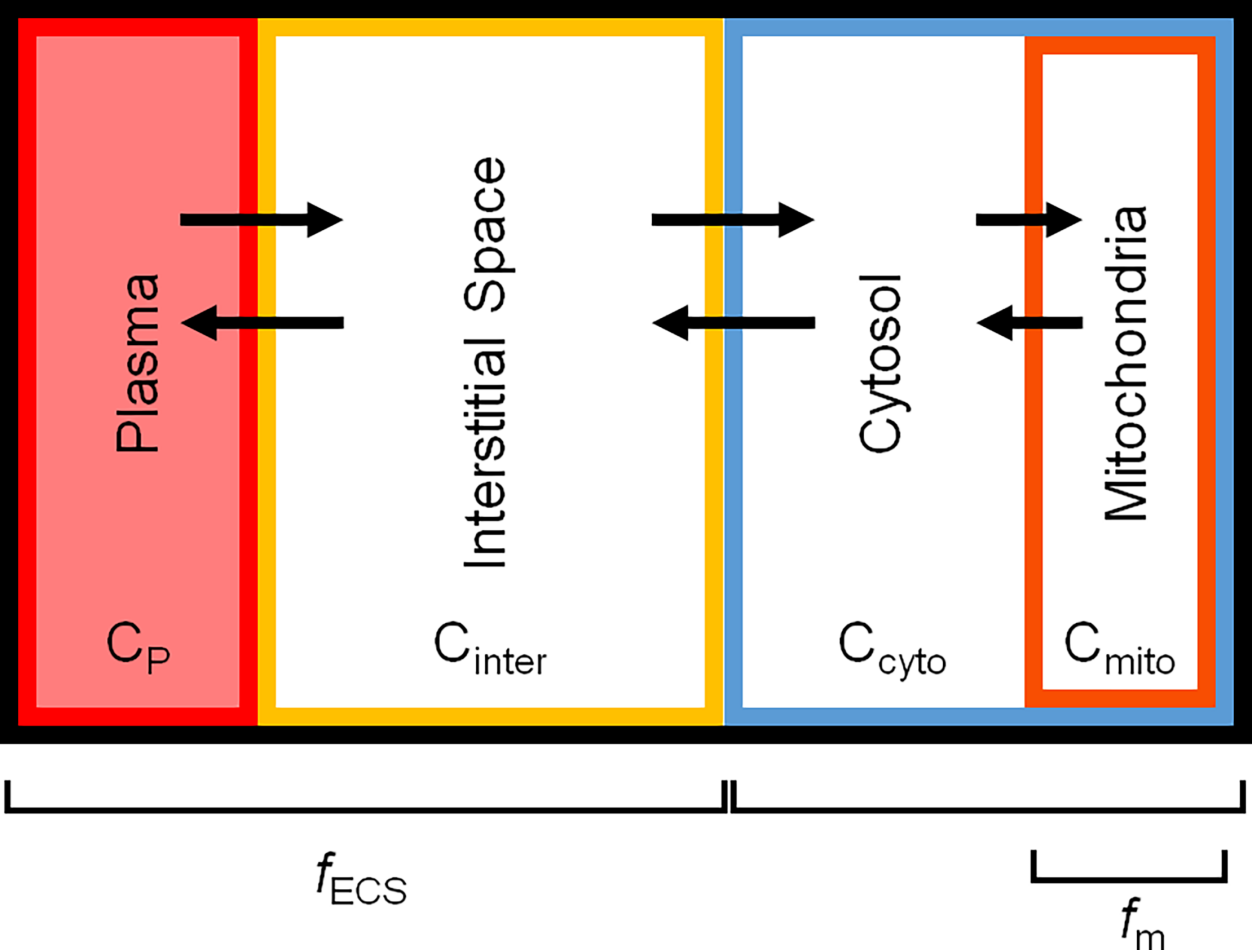
Volume of distribution model for ^18^F-TPP^+^ in a PET image voxel. The outer black line represents the voxel boundary. C_p_, C_inter_, C_cyto_, and C_mito_ represent the concentrations of the plasma, interstitial space, cytosol, and mitochondria respectively. The arrows represent ^18^F-TPP^+^ transport between the different compartments. *f*_ECS_ represents the voxel volume fraction occupied by ECS and *f*_mito_ represents the cellular volume fraction occupied by mitochondria.
